# Oral Diagnostic Methods for the Detection of Periodontal Disease

**DOI:** 10.3390/diagnostics11030571

**Published:** 2021-03-22

**Authors:** Liza L. Ramenzoni, Marc P. Lehner, Manuela E. Kaufmann, Daniel Wiedemeier, Thomas Attin, Patrick R. Schmidlin

**Affiliations:** 1 Clinic of Conservative and Preventive Dentistry, Center of Dental Medicine, University of Zurich, 8032 Zurich, Switzerland; info@drlehner.ch (M.P.L.); manuela.kaufmann@zzm.uzh.ch (M.E.K.); thomas.attin@zzm.uzh.ch (T.A.); 2 Laboratory of Applied Periodontal and Peri-Implantitis Sciences, Clinic of Conservative and Preventive Dentistry, Center of Dental Medicine, University of Zurich, 8032 Zurich, Switzerland; 3 Statistical Services, Center of Dental Medicine, University of Zurich, 8032 Zurich, Switzerland; daniel.wiedemeier@zzm.uzh.ch

**Keywords:** saliva, point-of-care diagnostics, periodontitis, biomarkers, oral disease

## Abstract

Periodontitis is a common immune-inflammatory oral disease. Early detection plays an important role in its prevention and progression. Saliva is a reliable medium that mirrors periodontal health and is easily obtainable for identifying periodontal biomarkers in point-of-care diagnostics. The aim of this study is to evaluate the effectiveness of diagnostic salivary tests to determine periodontal status. Whole saliva (stimulated/unstimulated) from twenty healthy and twenty stage III grade B generalized periodontitis patients was tested for lactoferrin, alkaline phosphatase, calcium, density, osmolarity, pH, phosphate, buffer capacity, salivary flow rate and dynamic viscosity. A semi-quantitative urinary strip test was used to evaluate markers of inflammation in saliva (erythrocytes, leukocytes, urobilinogen, nitrite, glucose, bilirubin, and ketones), clinical periodontal parameters and pathogenic bacteria. Concentrations of lactoferrin, hemoglobin, and leukocytes were found to be significantly higher in the stimulated and unstimulated saliva in periodontitis patients compared to healthy patients, whereas alkaline phosphatase levels were higher in unstimulated saliva of periodontitis patients (*p* < 0.05). Periodontal biomarker analysis using test strips may be considered rapid and easy tool for distinguishing between periodontitis and healthy patients. The increase in lactoferrin, hemoglobin, and leucocytes—determined by strip tests—may provide a non-invasive method of periodontal diagnosis.

## 1. Introduction

Periodontitis is considered one of the most prevalent immune-inflammatory diseases of the oral cavity. It derives from a specific pathogenic bacteria–host interaction and leads to periodontal tissue destruction [1,2]. The progression of periodontitis is often characterized by irregular phases of increased activity and dormant remission [3,4,5]. Traditional clinical periodontal assessment methods, such as pocket probing depth (PPD), bleeding on probing (BOP), clinical attachment level (CAL), and radiological assessment of the alveolar bone volume, are widely used and documented [1,2]. However, these traditional periodontal classification parameters fail to provide noteworthy information on current disease activity, severity and extent of breakdown, future progression and therapy response [2,6]. More importantly, the biological phenotype of the patient is not properly reflected by the clinical assessment methods [7] and the host response to periodontal bacteria and the subsequent inflammatory burden, i.e., the influence of biological phenotype, may largely determine periodontitis progression. Further, an early diagnosis may lead to more successful treatment [8,9]. In an attempt to redesign the periodontal disease framework, the new periodontal classification includes multidimensional staging and grading system parameters, which allows one to partially assess the future periodontal risk [2,8]. Although the new grading risk factors, i.e., smoking and diabetes, are included to predict the likelihood of future periodontal breakdown, they are still not able to determine the exact time of disease manifestation [2,8]. Thus, adding more robust biomarkers to the new classification system will improve the identification of active periods of periodontitis, monitor progression and avoid consequent mismanagement of periodontal treatment.

To better clinically identify the biological progression or phases of periodontitis within the new classification system, the development of new diagnostic tests is still required. Periodontitis is closely associated with tempering specific immune responses, and its modifying factors are typically found in the gingival crevicular fluid (GCF) and saliva during progression of the disease [2,10]. Oral fluid-based point-of-care diagnostics have been previously documented as potential chairside tests for determining periodontal diseases [2,11]. In order to measure disease activity, samples of saliva and GCF have been shown to mirror the periodontal condition and may be reliable mediums for biomarker detection [12]. Saliva offers many advantages in point-of-care diagnostics as it is easily obtainable, may be collected non-invasively and is rich in more than a thousand different diagnostic biomarker molecules for chronic inflammation and tissue destruction [13,14]. In addition, many compounds found in the blood are also found in saliva. Thus, saliva is a very useful tool for monitoring not only oral, but also systemic health [15]. In this respect, several molecular approaches, such as PCR for RNA/DNA or ELISAs for proteins, have been proposed for detecting periodontal biomarker molecules in saliva using advanced laboratorial techniques [14,16]. However, subject to further study, simpler and more easily applicable salivary techniques may provide the pathophysiological parameters necessary for a diagnosis of periodontitis and obviate the need for blood samples or histological sections, which are traditionally required. There is also a lack of simple and affordable methods to identify biomarkers that may both diagnose the disease and predict the risk of future disease activity.

The aim of this study is to evaluate a simple, well-established, and cost-effective urine analysis test to indicate and diagnose periodontitis. A semi-quantitative strip test that has not previously been applied in oral studies was used in an attempt to easily identify and distinguish between six salivary biomarkers in periodontitis patients as compared to healthy patients. In addition, differences in salivary composition, both in a stimulated and unstimulated state, were analyzed. We hypothesized that a strip test traditionally used for urine analyses may perform equally well as a diagnostic test for periodontal disease biomarkers and aid the determination of periodontal inflammatory severity (stage) and risk (grade) in periodontitis patients.

## 2. Materials and Methods

### 2.1. Participants and Study Design

A total of 40 patients (24 men and 16 women) referred for treatment to the Clinic of Conservative and Preventive Dentistry, Center of Dental Medicine, University of Zurich agreed to participate in this study. The clinical considerations for periodontitis patients were made following the new classification of 2018 [2]. Half of the participants were recruited from patients classified with the new periodontitis classification system [2] who presented generalized periodontitis stage III (interdental CAL ≥ 5 mm, radiographic bone loss extending to middle third of root and beyond, loss of 4–5 teeth due to periodontitis) and grade B progression (radiographic bone loss < 2 mm over 5 years, half pack or less per day smoking, biofilm commensurate with destruction). Periodontitis patients also presented PPD ≥ 6 mm, vertical bone loss ≥ 3 mm, furcation involvement class II or III, moderate ridge defects [2]. The remaining 20 individuals were periodontally healthy (PPD ≤ 3 mm) with no CAL or BOP (≤10%), and no signs of gingival inflammation (redness, clinical swelling, edema or pain) [2]. All patients were comprehensively informed about the content and purpose of the study before becoming involved. They were also informed about the irreversible anonymization and destruction of the samples, their right to withdraw from the study at any time and the consequences of anonymization according to Art. 30 HFV (Swiss Human Research Act). The study protocol was approved by the Ethics Committee of the Canton of Zurich (BASEC-Nr. 2018-00221) in accordance with the Helsinki Declaration. As regards the sample size calculation, a prevalence of 0.5% was employed as no previous data were available in the literature. The confidence interval was set at 95% by accepting a margin of error of 10%. Given these variables, 40 participants were required. By reducing the margin of error and increasing the accuracy, the sample number increased according to the usual parameters.

The inclusion criteria for the periodontitis patients were males or females, smokers and non-smokers between 35 and 60 years of age, with stage III grade B generalized periodontal disease, and an otherwise good general state of health with no grade modifier on systemic risk factors (i.e., no caries, no diabetes, no HIV-positive status) [2]. The inclusion criteria for healthy patients were the aforementioned age range, no PPD ≥ 3 mm, no CAL, BOP (≤10%), or gingival inflammation [2]. The exclusion criteria for both test groups were as follows: pregnant or those in lactation, patients who had been administered antibiotics and/or anti-inflammatory medication within the last 6 months and/or received periodontitis treatment within the last 2 years.

### 2.2. Clinical Evaluation

All clinical parameters and anamneses of medical and dental history/periodontal status for all study participants were taken by one calibrated examiner (M.E.K.). The evaluation included the following clinical parameters: decayed, missing, and filled teeth; tilt or overeruption; mobility and sensitivity; clinical attachment loss (CAL), PPD, and BOP [2,17]. The periodontal pockets were measured using a manual probe (Deppeler SA, CH-1180 Rolle, Switzerland) at six points circularly around each tooth. The presence or absence of pus secretion, the presence or absence of gingival recession, the presence or absence of furcation, and the presence or absence of plaque were recorded. If available, pre-existing X-rays not older than 2 years were used. In the periodontitis group, an interdental CAL ≥ 5 mm with radiographic bone loss extending to middle third of root and ≤ 4 teeth lost due to periodontitis were considered stage III periodontitis [2]. The calculation of periodontal inflamed surface area (PISA) as the percentage of overall periodontal tissue was based on the clinical parameters CAL and PPD [18].

### 2.3. Sample Collection

The study participants were instructed to avoid eating, drinking, or brushing their teeth 1 h prior to saliva sample collection. First, unstimulated saliva was periodically expectorated into disposable collection tubes (Polystyrol PS, 30 mL, Semadeni Plastics Group, Ostermundigen, Switzerland) for a period of 15 min [19]. Subsequently, the patients were asked to chew on a piece of parafilm (Bemis Company Inc. Oshkosh, WI, USA) for approximately 5 min until stimulated saliva was collected. The saliva samples were weighed and the salivary flow rate (mL/min) was calculated for further analysis. Finally, RNA-based microbiological sampling was executed with microbiological testing (Pado Test, Institute for Applied Immunology IAI AG, Zuchwil, Switzerland). All samples for each patient were pooled and sent to an external laboratory to determine the presence (or absence) of the bacterial markers *Aggregatibacter actinomycetemcomitans (Aa), Tannerella forsythia (Tf), Porphyromonas gingivalis (Pg), Treponema denticola (Td), Prevotella intermedia (Pi)* and *Filifactor alocis (Fa)* [10,20].

### 2.4. Saliva Analysis

**Figure 1:**
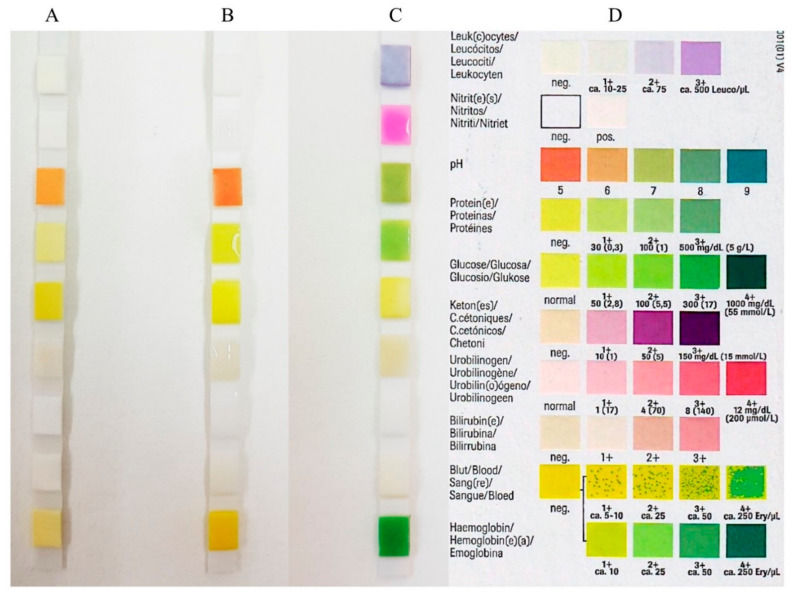
Reagent strip (Combur9-Test Cobas test strips) performed on saliva samples in order to determine: pH, leukocytes, erythrocytes, levels of nitrites, proteins, glucose, ketone bodies, urobilinogen, and bilirubin. (**A**) Blank test strip; (**B**) control with 10 µL water/test field each; (**C**) saliva sample 10 µL/test field each; (**D**) color reference.

Before investigation, stimulated and unstimulated saliva samples were centrifuged for 10 min at 6000 rpm at 4 °C and the supernatants were stored in new 2 mL tubes (Eppendorf AG, Schönbuch, Switzerland) at −80 °C [21]. Salivary osmolarity (mOsm/L) was measured using a micro-osmometer (Fiske Model 210 Micro-osmometer, K. Schneider & Co. AG, Zurich, Switzerland). Calibration was performed using the Gerber osmolarity standard 300 mOsm/kg H_2_O for a result of 300 mOsm/L saliva. In addition, the pH value of the saliva specimens was analyzed (Metrohm, Herisau, Switzerland). The pH meter was calibrated before the measurements with two buffer solutions of pH 7.00 and pH 4.00 (Thermo Fisher Scientific AG, Reinach, Switzerland). Moreover, the buffer capacity of the saliva (mL/0.1 M HCl) was determined by titration with 0.1 M/L HCl solution (PanReac AppliChem, Darmstadt, Germany). The buffer capacity corresponded to the consumption of 0.1 M/L HCl solution up to a pH of 5.7. Phosphate determination (mmol/L) was performed with the spectrophotometer (Portmann Instruments AG, Biel-Benken, Switzerland) at 750 nm. The obtained data were multiplied by 100 to determine the phosphate concentration in g P/mL saliva. Calcium (Ca (mmol/L)) was also determined with a spectrophotometer at 422 nm (Analytic Jena AG, Jena, Germany). Finally, the dynamic viscosity (mPa.s) and density (g/cm^3^) of samples was investigated with a micro-viscometer (Lovis 2000 M/ME, Anton Paar GmbH, Graz, Austria). The determination was carried out at 20 °C according to the specifications of Anton Paar GmbH. A reagent strip Combur9-Test Cobas (Roche Diagnostics GmbH, Mannheim, Germany) was performed on saliva samples to determine pH, leukocytes, erythrocytes, levels of nitrites, proteins, glucose, ketone bodies, urobilinogen, and bilirubin. The procedures were performed according to manufacturer’s instructions. The test strips were moistened at room temperature with 10 μL of unstimulated saliva and 10 μL of stimulated saliva from samples from each participant. After 1 min, a visual check for a possible color change was conducted and the color reference indicated by individual tests was used as the basis for comparison (Figure 1). The most clearly visually recognizable color change compared to the manufacturer’s color reference was registered. The semi-quantitative parameters determined by the test included the following: (1) pH (5/6/7/8/9); (2) leucocytes (negative/10-25/75/500 leucocytes/µL); (3) nitrite (negative/positive); (4) protein (negative/30/100/500 mg/dL); (5) glucose (50/100/300/1000 mg/dL); (6) ketone bodies (negative/10/50/150 mg/dL); (7) urobilinogen (normal/1/4/8/12 mg/dL); (8) bilirubin (negative/1+/2+/3+); and (9) hemoglobin/hemolyzed erythrocytes (negative/10/25/50/250Ery/µL).

### 2.5. Alkaline Phosphatase and Lactoferrin Analysis of Saliva

Alkaline phosphatase (mU/mL) was detected using a kinetic color test (ab83371, Abcam, Alkaline Phosphatase Assay Kit, Cambridge, UK) and a 96-well plate (Thermo-fisher Scientific/Life Technologies, Waltham, MA, USA) at 450 nm in a spectrophotometer (Bucher Biotec AG, Basel, Switzerland). Lactoferrin (μg/mL) was analyzed with ELISA (enzyme-linked immunosorbent assay kit, Abcam, Cambridge, UK) following a previously described protocol [21]. The absorbance at 450 nm was recorded for each ELISA on a microplate reader (EZ Read 400 Microplate Reader; Biochrom, Cambourne, UK) and the absorbance reference value (540 or 570 nm) was subtracted from the test values. Experiments were performed on three specimens from each test group in order to confirm the dilution factor of each biomarker. All experiments were conducted in triplicate.

### 2.6. Statistical Analysis

The data were analyzed and plotted using the statistical software R (The R Foundation for Statistical Computing, Vienna, Austria) and Microsoft Excel for Mac (v16.37, 20051002, Microsoft Corporation, Redmond, WA, USA). The statistical evaluation of all parameters in Table 1, Table 2 and Table 3 was performed using the Wilcoxon rank-sum test, with the exception of the binary data for nitrite, which was tested using Fisher’s exact test. The statistical significance level was set at α ≤ 0.05.

## 3. Results

### 3.1. Clinical Evaluation and Sample Collection

The descriptive statistical analysis for all participants is listed in Table 1. In brief, this study evaluated forty patients in total aged between 35 to 60 years old; twenty participants had stage III grade B generalized periodontal disease and twenty were periodontally healthy participants. For the periodontal patients, the mean collective PPD was 4.24 mm, with a range of 5–12 mm. The healthy patients had a mean collective sulci depth of 2.01 mm, with a range of 2–3 mm. Next, clinical inflammation values were determined by recording the BOP and by calculating the periodontal inflamed surface area (PISA). The healthy patients exhibited limited BOP with a mean clinical attachment level (CAL) of 1.86 mm. The difference between the highest and the lowest range values was calculated and BOP was found to be lower in healthy patients, with an interquartile range (IQR) of 9 in comparison to an IQR of 28 for the periodontitis patients. As expected, periodontitis patients exhibited increased BOP with a higher mean CAL of 3.45 mm. The IQR PISA value for the healthy patients was 366, while the IQR value for the periodontitis patients was 2112, hence, almost six times greater. Both BOP and PISA were significantly higher for the periodontitis patients compared to the healthy patients (*p* < 0.001).

### 3.2. Salivary Analysis

The salivary parameters for unstimulated and stimulated saliva are given in Table 2. Within the unstimulated saliva samples, a significantly higher volume (*p* = 0.003) and higher salivary flow rate (*p* = 0.002) were found for the healthy patients. Results from a comparison of stimulated saliva between the healthy and periodontitis patients were not statistically significant for volume and salivary flow rate. The level of alkaline phosphatase in unstimulated saliva from periodontitis patients was significantly higher than that of the healthy patients (*p* = 0.03). No significance was found for these levels in stimulated saliva (*p* = 0.2). The osmolarity showed significantly higher levels for periodontitis patients in both unstimulated (*p* < 0.001) and stimulated saliva (*p* = 0.006).

**Figure 2:**
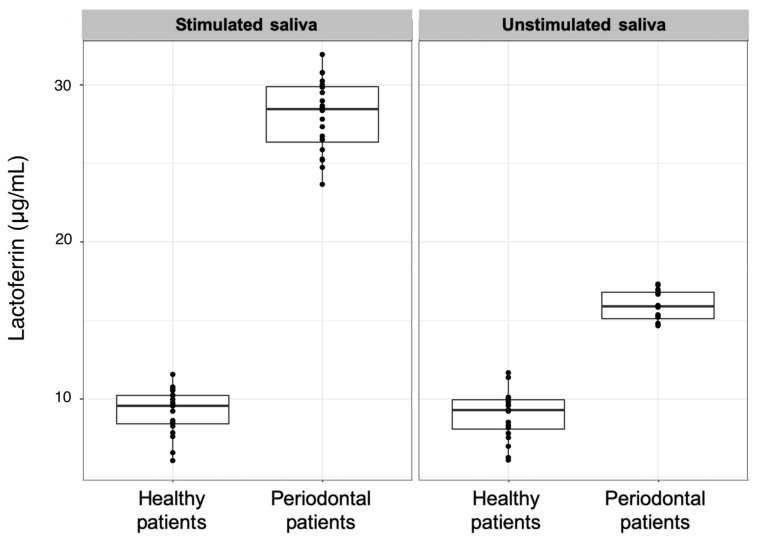
Comparison between lactoferrin measurements in stimulated and unstimulated saliva samples from healthy and stage III grade B generalized periodontal patients.

Lactoferrin was the most distinct parameter between the healthy and periodontitis patients stage III grade B in both unstimulated and stimulated saliva (Table 3); it was found to be two to three times higher in periodontitis patients compared to healthy patients (Figure 2).

**Figure 3:**
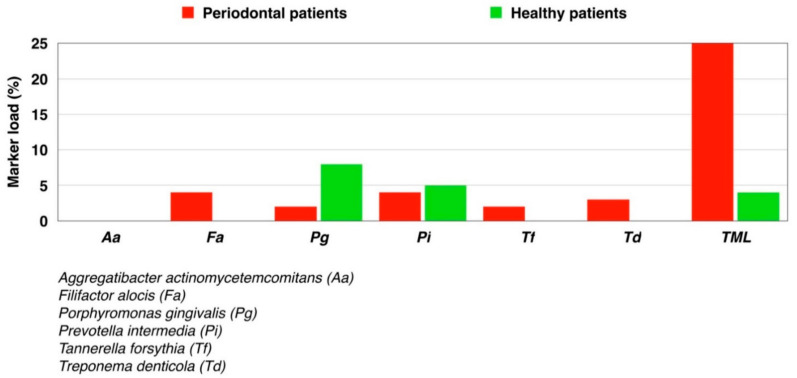
Marker load (%) of known pathogenic markers for periodontal bacterial species tested in healthy and periodontal patients. TML: Total marker load.

For the other salivary parameters—buffer capacity, calcium, density, dynamic viscosity, pH, and phosphate—no significant difference was found between stimulated or unstimulated saliva of healthy or stage III grade B periodontitis patients. Regarding the microbiological test, the only bacterium not detected in any of the samples was *Aa*. In contrast, *Tf, Pg, Td, Pi,* and *Fa* were all detected in small quantities, with a detection limit of < 0.3% (Figure 3).

The evaluation of salivary inflammation was determined using a urine reagent strip Combur 9-Test Cobas to identify bilirubin, erythrocytes, glucose, hemoglobin, ketones, leukocytes, nitrite, pH, protein and urobilinogen (Table 3). No bilirubin, glucose, ketones, or urobilinogen was detected in the unstimulated or stimulated saliva. Hemoglobin, as a component for blood determination in urine, was detected in the stimulated and unstimulated saliva of both healthy and stage III grade B periodontitis patients. In addition, hemoglobin was found to be higher in periodontitis patients compared to healthy patients in both unstimulated (*p* = 0.0007) and stimulated saliva (*p* = 0.006). In the leukocyte measurement, a significant increase was also detected for periodontitis patients in both stimulated (*p* = 0.002) and unstimulated (*p* = 0.002) saliva. Nitrite values were found to be positive for both unstimulated and stimulated saliva, without significant differences between healthy and periodontal patients. The pH parameter only showed a significant increase in the unstimulated saliva, which was the case for both healthy and periodontitis patients (*p* = 0.01). Furthermore, the pH of stimulated saliva was slightly more basic (pH = 8) than unstimulated saliva (pH = 7). Protein detection was positive for all study participants, yet with no significant difference between stimulated (*p* = 0.3) and unstimulated (*p* = 0.9) saliva in healthy and periodontal patients.

## 4. Discussion

Newly developed point-of-care periodontal diagnostic tests led us to infer that there may be a better means of diagnosing periodontitis and assessing its stage activity than the traditional clinical assessment methods [22,23,24,25]. The main purpose of this pilot study was to determine whether a commercially available, rapid and inexpensive urine test strip could be used to determine salivary inflammation parameters in stage III grade B generalized periodontitis patients. To our knowledge, urine reagent strip tests so far have not been evaluated with saliva for testing for periodontal inflammation indicators. Our findings showed that saliva is a suitable medium to identify inflammation by using fast and cost-effective urine test strips. The urine semi-quantitative diagnostic test was chosen for testing in this study, as its results are both available within 1 min and it has a user-friendly color scale for evaluation of the results. In comparison with point-of-care diagnostic tests, traditional parameters, such as BOP and PPD, have been suggested as major clinical measurements within the old periodontal classification to determine on-going periodontal disease. Nevertheless, BOP and PPD alone fail to predict future periodontal breakdown. Although the new classification system uses 10% of BOP as a cut-off to define periodontal health, the risk of false positive diagnosis could be increased by possible overlapping among stages and grades of patients [22,23,24,25]. Within the various point-of-care tests, urine test strips have proven to be effective over time in medical institutions and are both inexpensive and easy to use at room temperature. In fact, the semi-quantitative rapid strip test was originally designed to recognize the presence of polymorphonuclear leukocytes and other abnormalities associated with infection in urine through the detection of enzyme leukocyte esterase [26]. Beyond its utility for urinary tract infection screening, the leukocyte esterase test has been evaluated, achieving variable results and reliability, for a great array of bacterial inflammatory diseases, including meningitis, peritonitis, peritoneal lavage in abdominal trauma, *Helicobacter pylori* in gastric mucosa, inflammatory synovial fluid, and vaginitis [26,27,28,29,30,31,32,33,34,35]. In addition, the urine strip test was developed for the analysis of nine different parameters, not all of which, however, are present in saliva. Accordingly, the parameters bilirubin, glucose, ketones, and urobilinogen are not detectable in saliva and were found to be negative in this study. Nevertheless, meaningful results of the test showed strikingly enhanced levels of hemoglobin and leukocytes in the periodontitis patients for stage III grade B, as compared to the healthy patients, in both unstimulated and stimulated saliva. One explanation for the increased numbers of leukocytes is that polymorphonuclear leukocytes are known to play a pivotal role in gingival inflammation, and the number of these cells may be increased in order to maintain oral health [36]. A previous study also showed a positive correlation between levels of leukocyte produced proteins, LFA-1 (lymphocyte-function-associated antigen-1) and ICAM-1 (intercellular adhesion molecule-1), with Stage III Grade C generalized periodontitis [37]. This correlation is confirmed in our results and corroborates the leukocyte evaluation as a reliable parameter to determine the pathogenesis and progression of more advanced stages of periodontal disease. In recent years, the quantification of polymorphonuclear leukocytes has been proposed as a screening tool for gingivitis, periodontitis and even systemic inflammation [37,38,39,40]. Regarding the presence of high levels of hemoglobin, one explanation is that, in saliva, it may be derived from the bleeding of the periodontal tissue. In this respect, the salivary hemoglobin levels in this study are in accordance with previous studies [41,42]. As shown here, hemoglobin and its iron content may also be a good candidate for assessing periodontal advanced stages, as increased iron levels were previously identified in untreated generalized periodontitis stage III–IV grade B–C and detected from subtle bleeding in inflamed gingival tissue associated with periodontal parameters and bone loss [40,41,42]. Hemoglobin salivary detection tests incorporated into dental check-ups could substantially assist early periodontitis diagnosis and help maintain oral health [43].

Regarding the other laboratory results, the values of alkaline phosphatase in unstimulated saliva were found to be higher in stage III grade B periodontitis patients as compared to healthy patients. Our findings are similar to those found by other authors, which presented high levels of salivary alkaline phosphatase in stage III–IV grade B–C periodontitis patients compared to the healthy control group [44]. Additionally, a marginally significant alkaline phosphatase difference could be observed in the unstimulated saliva of healthy and stage III grade B periodontitis patients. Under stimulated conditions, only a tendency was noted, which could be due to the high dilution level. The difference was not significant. Alkaline phosphatase is a homodimeric cellular protein enzyme that may serve as an indicator of extensive cell damage and can be detected in elevated concentrations in the presence of inflammation and prevalent tissue destruction [45]. The higher level of alkaline phosphatase present in stage III grade B generalized periodontitis patients in this study confirms that it could be a valuable parameter to identify periodontitis. Furthermore, alkaline phosphatase is a well-known indicator of inflammation and cell damage in chronic periodontitis [45], with reduced levels being found in healthy patients and in patients after periodontal therapy [46]. Another key periodontal parameter used in this study was lactoferrin. The results showed significantly elevated levels of lactoferrin in both the unstimulated and stimulated saliva of stage III grade B periodontitis patients, as compared to the lower concentrations found in healthy patients. In fact, periodontitis patients were clearly distinguishable from healthy patients based on lactoferrin measurements alone. Furthermore, the range of lactoferrin measured in the present study is similar to that observed in a previous study on advanced stages of periodontitis patients [21]. Lactoferrin is secreted by myeloid and secretory epithelial cells. One feature of lactoferrin is its ability to bind iron, which removes one of the important elements involved in oral bacterial cell growth from saliva [47]. Therefore, lactoferrin is purported to have antibacterial activity [48]. The effect of unsaturated lactoferrin is greater than that of lactoferrin, which is fully saturated with iron. This fact is also evident in patients with stage III grade B generalized periodontal disease, i.e., the antibacterial activity of lactoferrin is lower in periodontitis patients than in healthy individuals [47,48]. As was found in this study, elevated levels and accumulation of lactoferrin are characteristics of patients with gingivitis [49] and periodontal disease [50,51]. Low concentrations of lactoferrin promote bacterial growth, as a result of the reduced antibacterial activity [51]. Among many other known point-of-care diagnostics biomarkers, increased levels of oral fluid matrix metalloproteinases, such as matrix metalloproteinases-8 (especially in activated/active form) are associated with periodontal and peri-implantitis diseases. Additionally, successful periodontal treatment has shown to halt progression of the disease, with a consequent reduction in matrix metalloproteinase-8 levels in the saliva [22,23,24,25]. Both qualitative and quantitative point-of-care immunotest technologies have been developed for the fast detection of pathologically elevated levels of active matrix metalloproteinase-8 in the saliva and may in fact assist in diagnosis and predict the prognosis of periodontitis [23]. Many reports have examined various different biomarkers in oral fluids in order to achieve diagnostic tools for periodontal disease, which have been designed into the latest classification system [23,52]. The majority of studies have focused on active MMP8 due to its importance. However, by also focusing on other biomarkers, such as lactoferrin, hemoglobin and leukocytes, as presented here, the increases in our understanding of the role of biomarkers in periodontal health and disease can be expected. This will lead to further point-of-care technology developments and consequently enable clinicians to diagnose periodontitis with prognosis prediction during therapy.

The final results of this study indicate the value of a rapid, cost-effective screening tool for periodontitis diagnostics. As discussed here, many periodontal salivary biomarkers have been suggested to be associated with periodontitis. However, some biomarkers, such as lactoferrin and alkaline phosphatase, require specialized testing capabilities and are mainly used in research. Additionally, the cost of measuring these specific biomarkers is still high, which further limits their application in routine clinical practice. However, the sensitivity and specificity of hemoglobin and leukocyte tests are higher than those of other periodontal biomarkers [41,53] and are significantly more reliable than information obtained using self-administered questionnaires for the screening of periodontal disease [54]. The further development of point-of-care periodontal diagnostic tests, such as for alkaline phosphatase or lactoferrin, may enable dental practitioners to measure inflammatory load using a rapid, non-invasive chairside approach, as opposed to relying on clinical parameters alone. Moreover, these tests could aid medical doctors in terms of assessing the periodontal status of patients with many different systemic diseases that are associated with periodontitis as grade modifiers, such as, endocarditis, atherosclerosis, and diabetes mellitus.

In conclusion, measuring hemoglobin and leukocyte levels in saliva using urine strip tests is less invasive than standard clinical periodontal assessments. Furthermore, saliva testing may be a viable alternative to the community periodontal index for periodontal screening. The analyses performed in this study show that the periodontal biomarkers lactoferrin, hemoglobin, and leukocytes are elevated in unstimulated and stimulated saliva in stage III grade B generalized periodontitis patients and their identification may complement current periodontal diagnostics. The development of a point-of-care test for lactoferrin would enlarge the diagnostic portfolio for periodontitis. The search for additional suitable parameters and the application of the data collected in this study for the evaluation of a new point-of-care test should be the focus of future studies.

## Figures and Tables

**Table 1:** Descriptive statistics of patient characteristics, clinical parameters, and a comparison of inflammation values of bleeding on probing (BOP), and periodontal inflamed surface area (PISA) as median (IQR) in healthy and periodontal patients (the statistical significance level was set at α ≤ 0.05).

Patient Characteristics	Healthy	Periodontitis Stage III Grade B
Age (mean ± SD)	50.47 ± 11.27	51.16 ± 12.35
Gender (m/f)	9/11	15/5
Smoking (yes/no)	5/15	12/8
**Clinical parameters**		
CAL^a^ (mean ± SD)	1.86 ± 0.23	3.45 ± 1.12
PPD ^b^ (mean ± SD)	2.01 ± 0.31	4.24 ± 1.35
BOP ^c^ (mean ± SD)	26.73 ± 22.96	28.59 ± 23.29
**Inflammation values**		
BOP ^c^ (%)	10 (9) ^e^	41 (28) ^e^
PISA ^d^ (mm^2^)	1010 (366) ^e^	2796 (2112) ^e^

^a^ CAL: clinical attachment level. ^b^ PPD: pocket probing depth. ^c^ BOP: bleeding on probing. ^d^ PISA: periodontal inflamed surface area calculated [18]. ^e^ *p*-value: < 0.001.

**Table 2:** Specific salivary parameters in median (IQR) obtained from stimulated and unstimulated saliva in healthy and periodontal patients.

	Unstimulated Saliva		Stimulated Saliva		Comparison	Comparison
	Healthy (A)	Periodontitis Stage III Grade B (B)	Healthy (C)	Periodontitis Stage III Grade B (D)	(A) vs. (B) (*p*-Value) ^a^	(C) vs. (D) (*p*-Value) ^a^
Sample volume (g)	5.7 (7.7)	2.2 (5.1)	5.2 (7.8)	4.7 (8.2)	0.003	0.7
Salivary flow rate ^b^ (mL/min)	0.51 (0.38)	0.34 (0.16)	1.67 (0.98)	1.64 (0.94)	0.002	0.6
Alkaline phosphatase (mU/mL)	0.97 (0.68)	2.46 (3.94)	0.64 (0.95)	0.90 (2.21)	0.03	0.2
Buffer capacity (mL 0.1 M HCl)	0.070 (0.022)	0.066 (0.053)	0.120 (0.061)	0.114 (0.064)	0.6	0.5
Calcium (mmol/L)	1.36 (0.30)	1.51 (0.68)	1.10 (0.19)	1.07 (0.20)	0.2	0.3
Density (g/cm^3^)	1 (0.00045)	1 (0.00015)	1 (0.00018)	1 (0.00090)	0.3	0.7
Dynamic viscosity (mPa·s)	1.276 (0.251)	1.117 (0.065)	1.107 (0.251)	1.085 (0.067)	0.1	0.3
Lactoferrin (µg/mL)	9.3 (1.9)	15.9 (1.7)	9.6 (1.8)	28.5 (3.5)	<0.001	<0.001
Osmolarity (mOsm/L)	66 (17)	98 (38)	71 (10)	76 (13)	<0.001	0.006
pH	7.43 (0.77)	7.19 (0.57)	7.97 (0.29)	7.95 (0.75)	0.1	0.4
Phosphate (mmol/L)	5.3 (3.1)	6.2 (3.2)	4.0 (1.3)	4.2 (1.5)	0.07	0.3

^a^*p*-value: statistical significance was set at *p* ≤ 0.05. ^b^ Saliva ml per minute: calculated value.

**Table 3:** Median (+/− IQR) of the saliva parameters bilirubin, erythrocytes, glucose, hemoglobin, ketones, leukocytes, nitrite, pH, protein and urobilinogen.

	Unstimulated Saliva		Stimulated Saliva		Comparison	Comparison
	Healthy (A)	Periodontitis Stage III Grade B (B)	Healthy (C)	Periodontitis Stage III Grade B (D)	(A) vs. (B) (*p*-Value) ^b^	(C) vs. (D) (*p*-Value) ^b^
Bilirubin (pos./neg.)	0 (0)	0 (0)	0 (0)	0 (0)	NA	NA
Glucose (mg/dL)	0 (0)	0 (0)	0 (0)	0 (0)	NA	NA
Hemoglobin (Ery/mL)	10 (25)	50 (225)	18 (25)	50 (225)	0.0007	0.006
Ketones (mg/dL)	0 (0)	0 (0)	0 (0)	0 (0)	NA	NA
Leukocytes (Leu/µL)	75 (0)	500 (425)	75 (425)	500 (0)	0.002	0.002
Nitrite ^a^ (pos./neg.)	(−)1/(+)19	(−)1/(+)19	(−)1/(+)19	(−)2/(+)18	NA	NA
pH	7 (1)	7 (1)	8 (0)	8 (0)	0.01	0.2
Protein (mg/dL)	65 (70)	65 (70)	30 (70)	30 (70)	0.9	0.3
Urobilinogen (mg/dL)	0 (0)	0 (0)	0 (0)	0 (0)	NA	NA

^a^ Nitrite: the evaluation of nitrites was performed using Fisher’s exact test. All other tests were performed using the Wilcoxon rank-sum test with continuity correction. ^b^ *p*-value: statistical significance was set at *p* ≤ 0.05.

## Data Availability

The data presented in this study are available on request from the corresponding author. The data are not publicly available due to ethical reasons.
